# The complete chloroplast genome sequence of *Helixanthera parasitica* (Loranthaceae)

**DOI:** 10.1080/23802359.2019.1660280

**Published:** 2019-09-06

**Authors:** Bingbing Liu, Yancai Shi, Ying Zhang

**Affiliations:** a Institute of Loess Plateau, Shanxi University, Taiyuan, China;; b Guangxi Key Laboratory of Plant Conservation and Restoration Ecology in Karst Terrain, Guangxi Institute of Botany, Guangxi Zhuang Autonomous Region and Chinese Academy of Sciences, Guilin, China

**Keywords:** *Helixanthera parasitica*, chloroplast genome, phylogenetic analysis

## Abstract

The complete chloroplast genome of *Helixanthera parasitica* from China was analyzed using next-generation sequencing. The plastome of *H. parasitica* is a typical quadripartite structure with a length of 125,021 bp, which contained inverted repeats (IR) of 22,752 bp separated by a large single-copy (LSC) and a small single copy (SSC) of 73,151 bp and 6,366 bp, respectively. The cpDNA contains 105 genes, comprising 67 protein-coding genes, 30 tRNA genes, and 8 rRNA genes. The overall GC content of the plastome is 36.5%. Phylogenetic analysis showed that *H. parasitica* was closely related to the *Tolypanthus maclurei*.

*Helixanthera parasitica* Lour., belonging to family Loranthaceae, is mainly distributed in tropical countries such as Thailand, Laos, Indonesia, Cambodia, Vietnam, and Philippines, as well as in south and southwest China. Recorded hosts for this species include *Castanopsis, Lithocarpus*, *Cinnamomum* and *Ficus* sp. (Romina and Daniel 2008). Here, we report and characterize the complete plastome of *H. parasitica* based on Illumina paired-end sequencing data, which will contribute to the further studies on its genetic research and resource utilization. The annotated cp genome of *H. parasitica* has been deposited into GenBank with the accession number MN080718.

In this study, *H. parasitica* was sampled from in Fujian province of China, located at 117°21′32.85″ E, 24°29′24.24″ N. A voucher specimen (Y.-C. Shi et al. H065) was deposited in the Guangxi Key Laboratory of Plant Conservation and Restoration Ecology in Karst Terrain, Guangxi Institute of Botany, Guangxi Zhuang Autonomous Region and Chinese Academy of Sciences, Guilin, China. The experiment procedure is as reported in Zhang et al. (2019). Around 2 Gb clean data were used for the cp genome de novo assembly by the program NOVOPlasty (Dierckxsens et al. 2017) and direct-viewing in Geneious R11 (Biomatters Ltd., Auckland, New Zealand). Annotation was performed with the program Plann (Huang and Cronk 2015) and Sequin (http://www.ncbi.nlm.nih.gov/).

The plastome of *H. parasitica* is a typical quadripartite structure with a length of 125,021 bp, which contained inverted repeats (IR) of 22,752 bp separated by a large single-copy (LSC) and a small single copy (SSC) of 73,151 bp and 6,366 bp, respectively. The cpDNA contains 105 genes, comprising 67 protein-coding genes, 30 tRNA genes, and 8 rRNA genes. Among the annotated genes, 6 of them contain one intron (*atp*F, *rpo*C1, *trn*L-UAA, *pet*B, *pet*D and *rpl*2), and three genes (*clp*P, *ycf*3 *and rps*12) contain two introns. The overall GC content of the plastome is 36.5%, which is unevenly distributed across the whole chloroplast genome.

To identify the phylogenetic position of *H. parasitica*, phylogenetic analysis was conducted. A neighbor-joining (NJ) tree with 1000 bootstrap replicates was inferred using MEGA version 7 (Kumar et al. 2016) from alignments created by the MAFFT (Katoh and Standley 2013) using plastid genomes of 14 species. It showed the position of *H. parasitica* was closely related to the *Tolypanthus maclurei* (Figure 1). Our findings will provide a foundation for facilitating its genetic research and contributing to its utilization in Loranthaceae.

**Figure 1.**
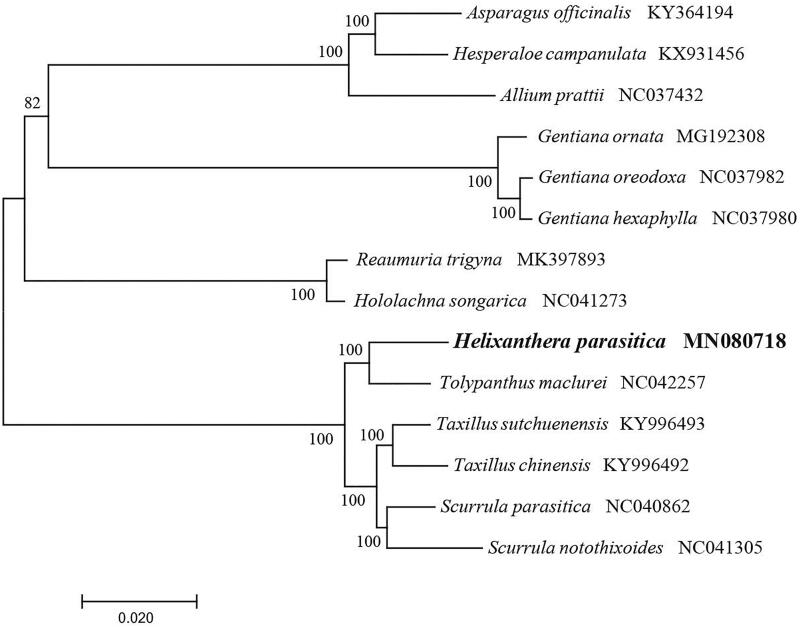
The Neighbour-Joining (NJ) tree based on the 14 chloroplast genomes. The bootstrap value based on 1000 replicates is shown on each node.
